# Neuropathological Changes in Nakalanga Syndrome—A Case Report

**DOI:** 10.3390/pathogens10020116

**Published:** 2021-01-23

**Authors:** An Hotterbeekx, Martin Lammens, Sylvester Onzivua, Robert Lukande, Francis Olwa, Samir Kumar-Singh, Stijn Van Hees, Richard Idro, Robert Colebunders

**Affiliations:** 1 Global Health Institute, University of Antwerp, 2100 Antwerp, Belgium; stijn.vanhees@uantwerpen.be (S.V.H.); robert.colebunders@uantwerpen.be (R.C.); 2 Molecular Pathology Group, Laboratory of Cell Biology & Histology, Faculty of Medicine and Health Sciences, University of Antwerp, 2100 Antwerp, Belgium; samir.kumarsingh@uantwerpen.be; 3 Department of Pathology, Antwerp University Hospital, 2100 Antwerp, Belgium; martin.lammens@uza.be; 4 Department of Neuropathology, Born-Bunge Institute, University of Antwerp, 2100 Antwerp, Belgium; 5 Department of Pathology, Makerere University Medical School, Kampala P.O. Box 7072, Uganda; sonzivua@gmail.com (S.O.); slylukas@gmail.com (R.L.); 6 Department of Diagnostics, Faculty of Health Sciences, Lira University, Lira P. O. Box 1035, Uganda; olwafrancis@gmail.com; 7 Translational Neurosciences, Faculty of Medicine and Health Sciences, University of Antwerp, 2100 Antwerp, Belgium; 8 Department of Pediatrics, Makerere University Medical School, Kampala P.O. Box 7072, Uganda; ridro1@gmail.com

**Keywords:** Nakalanga syndrome, nodding syndrome, epilepsy, post-mortem, pituitary gland, Uganda

## Abstract

Nakalanga syndrome is a clinical manifestation of onchocerciasis-associated epilepsy characterized by stunting, delayed or absent secondary sexual development and skeletal deformities, and is often accompanied by epileptic seizures. The pathophysiology of Nakalanga syndrome is unknown. Here, we describe the post-mortem findings of a 17-year-old female who died with Nakalanga syndrome in northern Uganda. Macroscopic and histopathological examination of all major organs (liver, lungs, kidney and heart), including the brain and the pituitary gland, was performed. The suspected cause of death was malaria, and all major organs and pituitary gland appeared normal, except the lungs, which were edematous consistent with the malaria. Neuropathological changes include signs of neuro-inflammation (gliosis and activated microglia), which co-localized with tau-reactive neurofibrillary tangles and threads. The pathology was most abundant in the frontal cortex, thalamic and hypothalamic regions, and mesencephalon. The choroid plexus showed psammoma bodies. These findings indicate accelerated aging, probably due to repeated seizures. The neuropathological findings were similar to other persons who died with onchocerciasis-associated epilepsy. Examination of the pituitary gland did not reveal new information concerning the underlying pathophysiological mechanism of Nakalanga syndrome. Therefore, more post-mortem studies should be performed.

The Nakalanga syndrome has been reported in many onchocerciasis-endemic regions, including South America [1], Uganda [2,3], Tanzania [4], Burundi [5], the Democratic Republic of Congo [6], and Cameroon [7]. The syndrome is characterized by stunted growth, delayed or absence of external signs of secondary sexual development, wasting, mental retardation, epilepsy, and thoracic and spinal abnormalities [3,5]. Nakalanga syndrome occurs in the same places where persons with epilepsy live who meet the criteria of onchocerciasis-associated epilepsy (OAE), and Nakalanga features are often overlapping with nodding seizures and other forms of OAE [2,8,9]. Epidemiological evidence has linked onchocerciasis with the Nakalanga syndrome [8], but the underlying pathophysiological mechanism remains unknown. Thus far, only one post-mortem exam has been performed in a person with Nakalanga syndrome in 1956, in a 30-year-old man who lived in the Mabira forest in Uganda [10]. His thyroid gland showed some evidence of hypofunction and his testes did not produce germ cells or the male sex hormone [10]. Examination of the pituitary gland showed few basophils but normal numbers of acidophils, which are not explaining the underlying growth retardation. Furthermore, an autopsy study on persons who died with nodding syndrome and other forms of OAE also included three individuals with a combination of nodding syndrome and Nakalanga features [11]. However, in these cases, only an extensive histopathological examination of the brain was performed, whereas the other organs were only investigated macroscopically and the pituitary gland was not taken [11]. Another autopsy study on persons who died with nodding syndrome identified tau-reactive neurofibrillary tangles and threads in the brain, but no information was available on potential Nakalanga features and the pituitary gland was also not investigated [12]. 

In this case report, we describe the post-mortem findings of a 17-year-old female with Nakalanga syndrome who died during an acute febrile illness in northern Uganda. A complete post-mortem exam was performed as before [11]. Samples taken from the visceral organs (lungs, liver, kidney and heart) and the complete brain were formalin fixed, embedded in paraffin (brain regions Table 1) and stained by haematoxylin-eosin. Peroxidase-based immunohistochemistry with haematoxylin counterstaining was performed to stain astrocytes (Polyclonal Rabbit Anti-Glial Fibrillary Acidic Protein), macrophages (Monoclonal Mouse Anti-Human CD68 KP1), and phosphorylated tau (AT8) as before [11]. Cresyl violet (Nissl staining), TDP-43, alpha-synuclein, Kluyver-barrera, Ubiquitin and p62 staining were performed on a selection of slides showing abnormalities, such as inclusions, pseudo-inclusions and vacuolizations in neuronal bodies. The pituitary gland was investigated for general abnormalities (Hematoxylin & Eosin, H&E) and immunohistochemistry of all pituitary hormones. Ethical approval was obtained from Lacor hospital, the Uganda National Council for Science and Technology (UNCST) and the ethics committee from the University hospital of Antwerp.

The deceased belonged to the Acholi tribe and lived in the Pader district of northern Uganda. She had an older sister who suffered from nodding syndrome. The deceased had head nodding seizures induced by the sight of food or by cold weather which started in 2005, at the age of 4 years. Several years later, the disease progressed to generalized tonic–clonic seizures and episodes of blank staring (absences) and wandering away from home were reported. There was no history of any severe disease, such as severe malaria or febrile illness, head trauma or abnormal development before the onset of the seizures, which could explain the development of seizures and the disease. Despite treatment with sodium valproate, carbamazepine and folic acid, three epileptic episodes weekly (nodding and generalized tonic–clonic seizures) were reported, as well as an episode of status epilepticus. She did not present onchocerciasis skin lesions or nodules, but she took ivermectin treatment during the bi-annual mass distributions in the region. 

Prior to death, the deceased was unable to perform normal household tasks, had a poor appetite and sometimes needed assistance with eating. She was very weak but able to walk without assistance, dress, wash and groom herself and was not bedridden. Around the time of death, she developed fever, headache and cough and was started on antimalarial treatment after a positive rapid diagnostic test. However, her clinical condition deteriorated and she was admitted to the hospital where she died three days later. 

External examination showed general wasting, with reduced subcutaneous fat and muscle mass, poor secondary sexual development, crippled legs, kyphosis and stunting (short stature). There was severe pallor, dehydration, mild jaundice and anemia but no palpable peripheral lymph nodes. Internal organs were normal, but smaller than expected for her age. The lungs were heavy and edematous with thick mucus secretions in the parenchyma and petechiae on the serous surfaces, which may have been related to the malaria infection prior to death [13]. 

Examination of the visceral organs (lungs, liver, kidney and heart) showed no abnormalities. Histological examination of the brain showed neuronal loss, especially the Purkinje cells and granular cells in the tops of the folia in the cerebellum. Spongiosis and an increased amount of glial cells were observed in the sub-pial layer at the top of the gyri of the left and right frontal cortex. Large neuronal bodies with inclusions, pseudo-inclusions and vacuolization were observed in the paraventricular nuclei on the right side (Figure 1A). The nature of these inclusions could not be determined by the additional staining performed. The choroid plexus showed strong fibrotic zones with strongly dilated blood vessels and psammoma bodies (Figure 1B). The ependymal layer of the right ventricle was interrupted locally, with some signs of gliosis and spongiosis. The hippocampus appeared normal in both the left and right side, with no signs of hippocampus sclerosis despite repeated seizures. Overlapping foci of tau-reactive neurofibrillary tangles, gliosis and activated microglia were observed as previously described in other OAE cases, although at a relatively higher abundance (Figure 1C). Tau-reactive tangles, threads and dots were found with variable abundance in almost all samples investigated, except for the choroid plexus and the left putamen (Table 1). The pathology was most abundant in the frontal cortex, thalamic and hypothalamic regions and mesencephalon (Table 1). The pituitary gland was normal (Figure 1D), with no signs of hormonal dysfunction. 

The pathophysiology of Nakalanga syndrome remains unknown. Two main characteristics of the syndrome include stunted growth and delayed or absence of secondary sexual development, pointing towards an endocrinological cause. Endocrinological investigations were performed earlier in eight persons with nodding syndrome in Uganda [14]. Here, serum hormone levels were normal in all but two individuals with severe stunting and one with moderate stunting, who had low levels of somatomedin C (insuline like growth factor (IGF1)) and/or IGF binding protein 3 (IGBP3), which are mediators of growth hormone function [14]. Furthermore, a linear relationship was observed between serum IGF1 and the height for age score [14]. 

In this case report, macroscopic investigation of the brain of the 17-year-old woman did not show any abnormalities; microscopic investigation of the brain revealed overlapping foci of tau-reactive neurofibrillary tangles, gliosis and activated microglia, as was previously observed in persons with OAE [11], further supporting the epidemiological evidence that Nakalanga syndrome is one of the clinical phenotypes of OAE. Furthermore, the abundance of tau-reactive neurofibrillary tangles, inclusions and psammoma bodies indicate accelerated aging [15], which might be associated with uncontrolled seizures [16,17]. 

One major drawback of post-mortem studies is that the disease is in its end stage, and factors underlying the pathological mechanism might no longer be present. However, the normal appearance of the pituitary gland in this case report and an earlier post-mortem study suggest that Nakalanga syndrome may not be caused by a structural dysfunction or lesion of the pituitary gland. Our post-mortem examination of the brain and pituitary gland did not provide further indication for the underlying pathophysiological mechanism. In agreement with Marshal and Cherry (1962), there was no evidence supporting an earlier suggestion that pituitary dysfunction in Nakalanga syndrome is the result of accumulating *O. volvulus* microfilariae [10]. Additional hormonal studies, such as pituitary stimulation tests, are needed to identify the pathophysiological mechanism. Moreover, as no height and no detailed Tanner scale [18] information was available in this patient, additional post-mortem studies on persons with clinically well-documented Nakalanga syndrome should be done. 

## Figures and Tables

**Figure 1:**
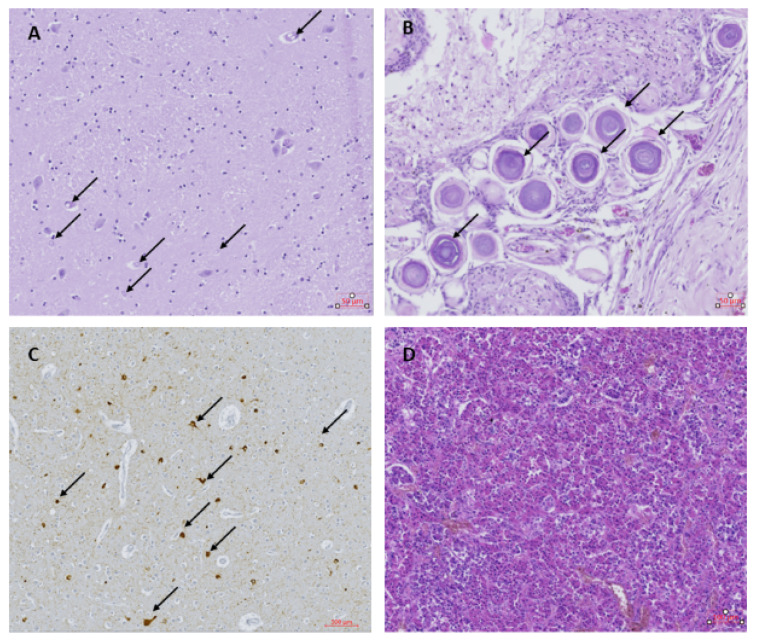
(**A**) Thalamus with inclusions and pseudo-inclusions (black arrows); Hematoxylin & Eosin (H&E) staining. (**B**) Choroid plexus containing psammoma bodies (black arrows); H&E. (**C**) Frontal cortex with tau-reactive neurofibrillary tangles and threads (black arrows); AT8. (**D**) Pituitary gland; H&E.

**Table 1:** Detailed histological distribution of gliosis (GFAP), activated macrophages (CD68) and tau-reactive neurofibrillary tangles (AT8).

Left Hemisphere				Right Hemisphere			
	GFAP	CD68	AT8		GFAP	CD68	AT8
Mesencephalon + substantia nigra	2+	±	2+	Olfactory nerve	+	±	2+
Pons	+	-	2+	Putamen + insular cortex	-	-	+
Pons + locus coeruleus	2+	+	2+	Putamen + pallidum + basal nucleus	+	±	2+
Cerebellum vermis	+	-	±	Putamen + insula + claustrum	±	-	+
Cerebellum hemisphere	+	-	±	Putamen + capsula interna	±	±	+
Cerebellum dentate nucleus	+	+	±	Basal temporal lobe	-	-	±
Cerebellum cortex	+	-	±	Amygdala	-	-	cortex±
Fronto-basal cortex	±	-	2+	Frontal cortex	2+	±	2+
Frontal cortex	+	-	2+	Fronto-basal cortex	2+	-	2+
Caudate nucleus	±	-	+	Caudate nucleus+ gyrus singuli	-	-	+
Corpus callosum + gyrus singuli	+	-	2+	Corpus callosum + gyrus singuli	+	±	+
Hippocampus (dentate gyrus)	-	-	±	Hippocampus	+	-	+
Hippocampus	+	-	+	Gyrus temporalis	+	-	2+
Thalamus	±	±	±	Thalamus + hypothalamus 1	+	+	2+
Putamen + insular cortex	-	-	-	Thalamus + hypothalamus 2	+	±	+
Temporal cortex + hippocampus anterior	±	-	±	Thalamus + paraventricular nuclei	+	±	+
Temporal lobe + hippocampus	-	-	+	Ventricle + capsula interna	-	-	±
Temporal lobe	+	-	+	Gyrus centralis	±	±	+
Primary visual cortex	2+	±	2+	Sulcus calcarinus	-	-	±
Parietal cortex	+	-	+	Parietal cortex	+	+	2+
Choroid plexus	-	+	-	Choroid plexus	-	-	-
